# Tryptophan and kynurenine levels in lenses of Wistar and accelerated-senescence OXYS rats

**Published:** 2009-12-16

**Authors:** Olga A. Snytnikova, Lyudmila V. Kopylova, Elena I. Chernyak, Sergey V. Morozov, Nataliya G. Kolosova, Yuri P. Tsentalovich

**Affiliations:** 1 International Tomography Center SB RAS, Institutskaya 3a, Novosibirsk, Russia; 2 Novosibirsk Institute of Organic Chemistry SB RAS, Acad. Lavrentjev 9, Novosibirsk, Russia; 3 Institute of Cytology and Genetics SB RAS, Acad. Lavrentjev 10, Novosibirsk, Russia

## Abstract

**Purpose:**

To determine the levels of kynurenine (KN) and its metabolic precursor tryptophan (Trp) in lenses of accelerated-senescence OXYS (cataract model) and Wistar (control) rats at ages from 1 day to 24 months.

**Methods:**

Protein-free lens extracts were prepared from Wistar and senescent-accelerated OXYS rat lenses. The presence and levels of KN and Trp were determined using high performance liquid chromatography (HPLC) analysis and mass spectrometric measurements. All statistical calculations were made using the software package Statistica 6.0, using factor dispersion analysis and Newman-Keuls post-hoc test for comparison of group mean values.

**Results:**

The levels of KN, which plays the role of a molecular Ultraviolet (UV) filter in the human lens, and its metabolic precursor Trp in the rat lens significantly depend on the rat strain and age. During the first 20 days after birth, before the first signs of cataract in OXYS rats, there is a strong difference in the content of both Trp and KN between Wistar and OXYS lenses. This difference becomes insignificant in lenses of 1 month and older. The levels of Trp and KN in young lenses are higher than that in lenses of 1 month and older for both strains.

**Conclusions:**

The presented results demonstrate that the KN pathway of Trp catabolism does not play a significant role in cataract development in the rat lens at the stages of cataract manifestation; however, in the first 3 weeks of postnatal development, the interstrain difference in KN and Trp levels is very strong. The obtained results show a correlation between the low level of KN and the high level of Trp at the stage of lens maturation and future cataractogenesis, and suggest an imbalance in the KN pathway of Trp catabolism in potentially cataractous lenses.

## Introduction

Cataract is the most frequent cause of impairment and loss of vision in elderly people [1]. The developed cataract is characterized by numerous posttranslational modifications of the major lens proteins, crystallins; these modifications include oxidation, cross-linking, truncation, and aggregation [2]. As a result of these changes, the lens proteins become colored, fluorescent, and insoluble [3,4]. Because there is no protein turnover in the lens core, the protein modifications accumulate with age, eventually resulting in cataract development. It is now commonly accepted that one of the major causes of protein modification is oxidative stress [2], in particular the imbalance between generation and detoxification of reactive oxygen species; however, the specific mechanisms of protein modification are still poorly understood. It has been suggested [2,5-8] that one of the possible mechanisms of protein modification is connected with thermal and/or photochemical reactions of ultraviolet (UV) filters—low-molecular-weight molecules contained in the lens and absorbing UV light in the 300–400 nm spectral region. Mammalian lenses contain a variety of such molecules, including kynurenine (KN) and its derivatives [6,9-16]. The covalent attachment of UV filters to the lens proteins may influence protein functionality and increase their susceptibility to UV light [17,18]. Indeed, it has been reported that with aging the level of free UV filters in the human lens decreases [19]; at the same time, crystallins modified by UV filters were found in aged human lenses [7,20-23]. Interestingly, the levels of both free and protein-bound UV filters in cataractous lenses are much lower than in normal lenses of the same age [20,22,24], whereas the level of the KN metabolic precursor tryptophan (Trp) is much higher [24]. This phenomenon can be attributed to the impairment in the Trp metabolic pathway in cataractous lenses as well as to the accelerated degradation of UV filters under oxidizing conditions. It has been suggested that protein modifications by KNs play an important role in cataract formation, but this has yet to be directly demonstrated. Study of the contribution of these processes in the etiology and pathogenesis of cataract in human lenses, especially at early preclinical stages, is almost an impracticable task.

The vast majority of experimental data on the biochemical content of cataractous human lenses corresponds to lenses with developed cataract surgically removed from patient eyes. The possibilities for studying early stages of cataract in humans are limited. One approach used for studies of etiology and pathogenesis of human diseases and for development of new methods for their treatment is the use of biological models. Among these models, rodents are by far the most widely used for cataract modeling: Emory mouse strain [25], the prone 9 of the senescence-accelerated mouse (SAM) [26], UPL Sprague–Dawley rats [27], and Shumiya cataract rats [28]. Recent studies have shown that the OXYS rat strain meets the main requirements for the model of senile cataract. The OXYS strain of rats was developed at the Institute of Cytology and Genetics, Russian Academy of Sciences (Novosibirsk, Russia), from Wistar stock by selection for their susceptibility to the cataractogenic effect of galactose [29]. Young Wistar rats were fed galactose-rich diets, and animals highly susceptible to the cataractogenic effect of this diet were selected for inbreeding. After five cycles of inbreeding, a galactose-rich diet, and selection, cataracts spontaneously developed in the following generations of rats without galactose in the diet. This rat strain was renamed the OXYS rat strain in 1996 by the International Rat Genetic Nomenclature Committee. At the present time the strain of OXYS rats is maintained under the guidance of one of the authors in the breeding experimental animal laboratory of the Institute of Cytology and Genetics.

The changes in the OXYS rat lens at clinical and morphological levels are similar to those in humans with developed senile cataract [30-32]. The clinical manifestation of the first signs of cataract in OXYS rats appears at the age of 1.5–2 months, and at the age of 3 months pathological changes have been found in 90% of animals [33]. Interestingly, an enhanced level of reduced glutathione (GSH) has been observed at early stages of cataract formation in OXYS rats [33]; this probably corresponds to a compensatory reaction to metabolic changes induced by oxidative stress. The model has been successfully used for estimation of the efficiency of new approaches to cataract treatment [34].

In the present study, we performed a comparative analysis of KN and Trp levels in lenses of OXYS and Wistar (which were used as control) rats of different ages. Because rats are nocturnal animals, it has been generally accepted that their lenses do not contain UV filters [2]. The presence of KN was found in lenses of transgenic mice but not in wild-type mice [35]. Very low concentrations of some KN derivatives (kynurenic acid, anthranilic acid, 3-hydroxykynurenine) in lenses of Wistar rats have been recently reported in the work of Raju et al. [36]. The present work demonstrates that the rat lens in fact contains KN (although at a significantly lower concentration than the human lens) and the levels of both KN and Trp strongly depend on the rat strain and age.

## Methods

### Materials and reagents

D,L-Kynurenine, L-tryptophan, N-acetyl-L-tryptophan, and trifluoroacetic acid were purchased from Sigma/Aldrich (St. Louis, MO) and used as received. H_2_O and ethanol were doubly distilled. Organic solvents (methanol and acetic acid; high performance liquid chromatography [HPLC] grade) were purchased from Cryochrom (St. Petersburg, Russia) and used as received.

### Sample preparation

All animal procedures adhered to the Association for Research in Vision and Ophthalmology statement for the Use of Animals in Ophthalmic and Vision Research and in compliance with the European Communities Council Directive No. 86/609/EES. Lenses for protein-free extraction were obtained from senescent-accelerated OXYS rats at 1, 7, 10, 15, and 20 days and 1, 1.5, 2, 3, 9, 14, and 24 months of age and from age-matched Wistar rats. The animals were housed in cages (45×35×35 cm) and kept under standard laboratory conditions (at 22±1 ºC, 60% relative humidity, natural light), provided with a standard rodent diet (PK-120-1; Laboratorsnab, Moscow, Russia), and given water ad libitum. Animals were decapitated. The lenses were removed, frozen in liquid nitrogen, and stored at -70 ºC until analysis. The extraction of UV filters was performed by homogenizing the lens in 80% ethanol (0.5 ml/lens pair). The homogenate was left on ice for 1 h and then centrifuged (15,000g, 30 min, 4 °C). The pellet was re-extracted with 80% ethanol (0.3 ml), and the combined supernatants were lyophilized, redissolved in 50 μl of water, then analyzed with the use of HPLC and Liquid Chromatography/Mass Spectrometry (LC/MS) methods.

### High performance liquid chromatography and mass spectrometry

HPLC analysis was performed with the use of an Agilent LC 1100 chromatograph (Agilent Technologies, Santa Clara, CA) equipped with a quaternary pump, an autosampler, and a diode array detector. Chromatographic conditions were as follows: ZORBAX Eclipse XBD-C8 column (4.6×150 mm, 5-μm particles); mobile phase consisting of methanol/0.1% (v/v) trifluoroacetic acid in H_2_O gradient; the methanol percentage in the gradient was 10–40% (0–15 min), 40% (15–20 min), 40–90% (20–25 min); flow rate was 0.9 ml/min; injection volume was 20 μl; the detection was performed simultaneously at five wavelengths: 254, 290, 314, 360, and 410 nm. Chromatograms were recorded and peak areas integrated with the use of Agilent ChemStation for Windows. Quantification of the KN and Trp levels in the lens extracts was performed with the use of the standard compounds KN and Trp.

LC/MS analysis was performed with an Agilent 1200 Series LC/MS instrument. The system includes a binary pump, a vacuum degasser, an autosampler, a thermostated column compartment, a diode array detector, and a mass-selective detector (micro Time Of Flight). Chromatographic conditions were as follows: ZORBAX Eclipse XBD-C8 column (2.1×50 mm, 3.5-μm particles); 30 °C; mobile phase consisting of 2.3% HCOOH/MeOH; the methanol percentage in the gradient was 10% (5 min), 10–50% (10 min), 50% (15 min); flow rate was 0.2 ml/min; injection volume was 4 μl.

The MS was performed with electrospray ionization (ESI) and atmospheric pressure chemical ionization (APCI) sources. MS conditions were the following: For ESI—Vcap 4,000 V, nebulizer pressure 30 psig, drying gas (N2) flow 10 l/min, drying gas temperature 340 °C, fragmenter 70 V, negative scan in the range m/z=140–800, positive scan in the range m/z=140–850; for APCI—Vcap 4000 V, nebulizer pressure 60 psig, drying gas (N2) flow 5 L/min, drying gas temperature 200 °C, vaporizer temperature 350 °C, fragmenter 70 V, negative and positive scans in the range m/z=140–800.

### Statistical analysis

All statistical calculations were made using the software package Statistica 6.0 (Statsoft, Tulsa, OK), using factor dispersion analysis (analysis of variance) and Newman-Keuls post-hoc test for comparison of group mean values. Genotype and age of animals were considered as independent factors. In all cases the results were considered as statistically significant at a p<0.05.

## Results

**Figure 1:**
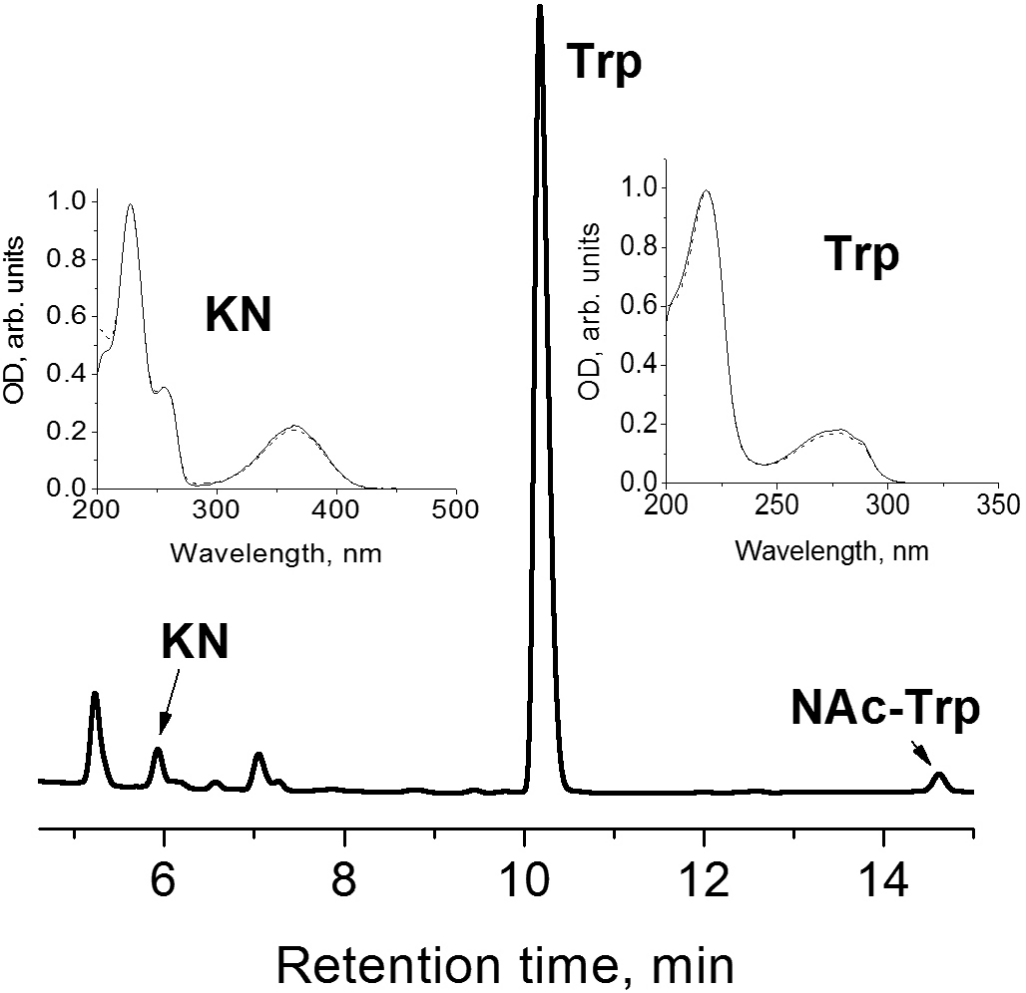
Chromatogram of protein-free extract from lenses of 15-day-old OXYS rat. The peaks marked by arrows are identified as kynurenine (KN), tryptophan (Trp), and N-acetyl-tryptophan (NAc-Trp). The inserts show absorption spectra of KN (5.9 min) and Trp (10.2 min) found in lenses, paired with the spectra of standard compounds (dashed lines).

Figure 1 shows the HPLC profile of the lens extract from a 15-day-old OXYS rat monitored at 254 nm. The signal eluted at 5.9 min has the same retention time and the same UV-Visible spectrum (Figure 1) as standard KN and a molecular weight of 208 Da. This signal was assigned to endogenous KN present in the rat lens. On a similar basis, the signals with the retention times of 10.2 min and 14.6 min were attributed to Trp and N-acetyl-tryptophan (NAc-Trp), respectively.

**Figure 2:**
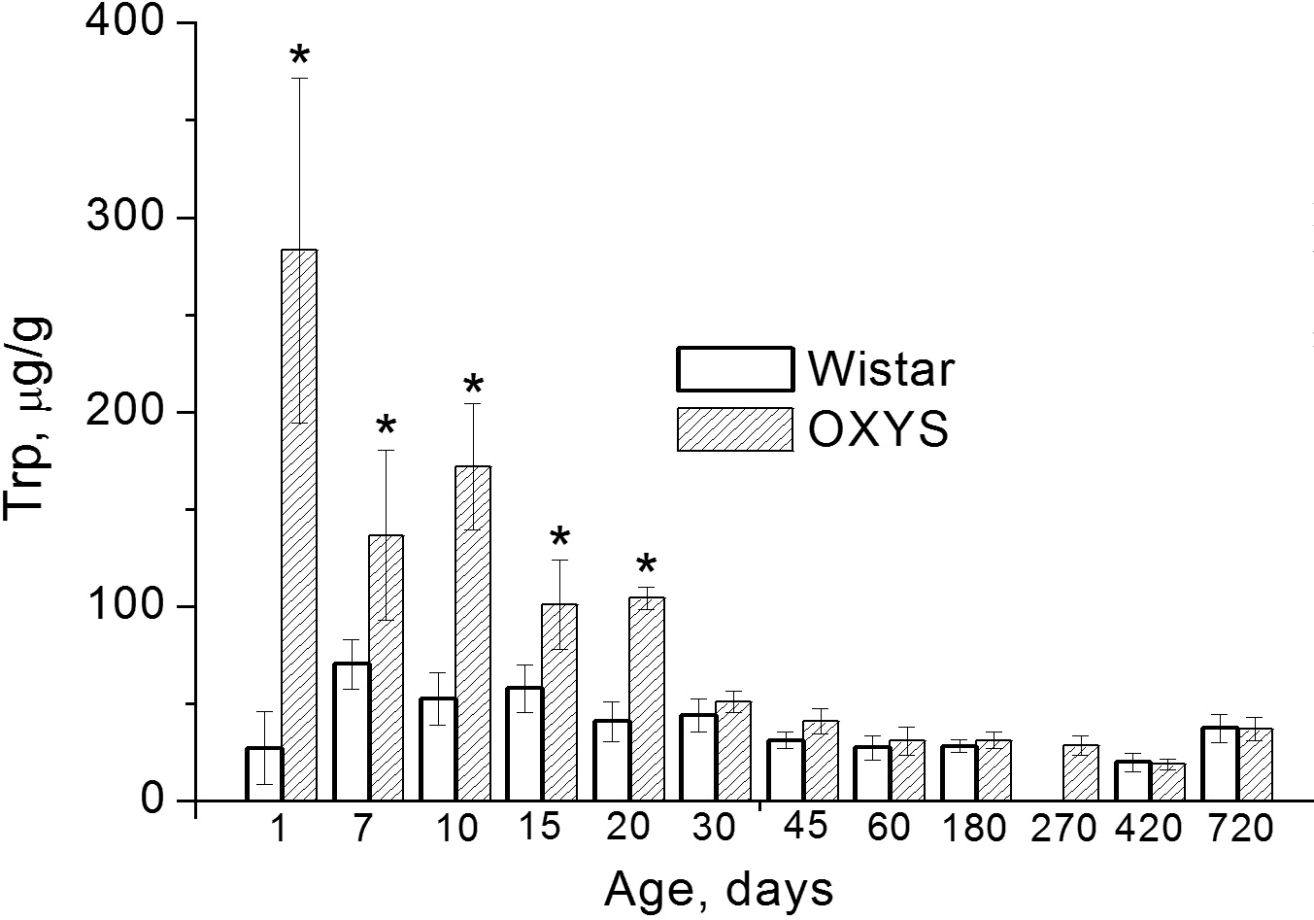
Depicted is the content of tryptophan (Trp) in lenses of Wistar (white bars) and OXYS (dashed bars) rats of different age. Error bars show standard deviations of Trp level in lenses. The asterisk indicates statistically significant interstrain differences.

**Figure 3:**
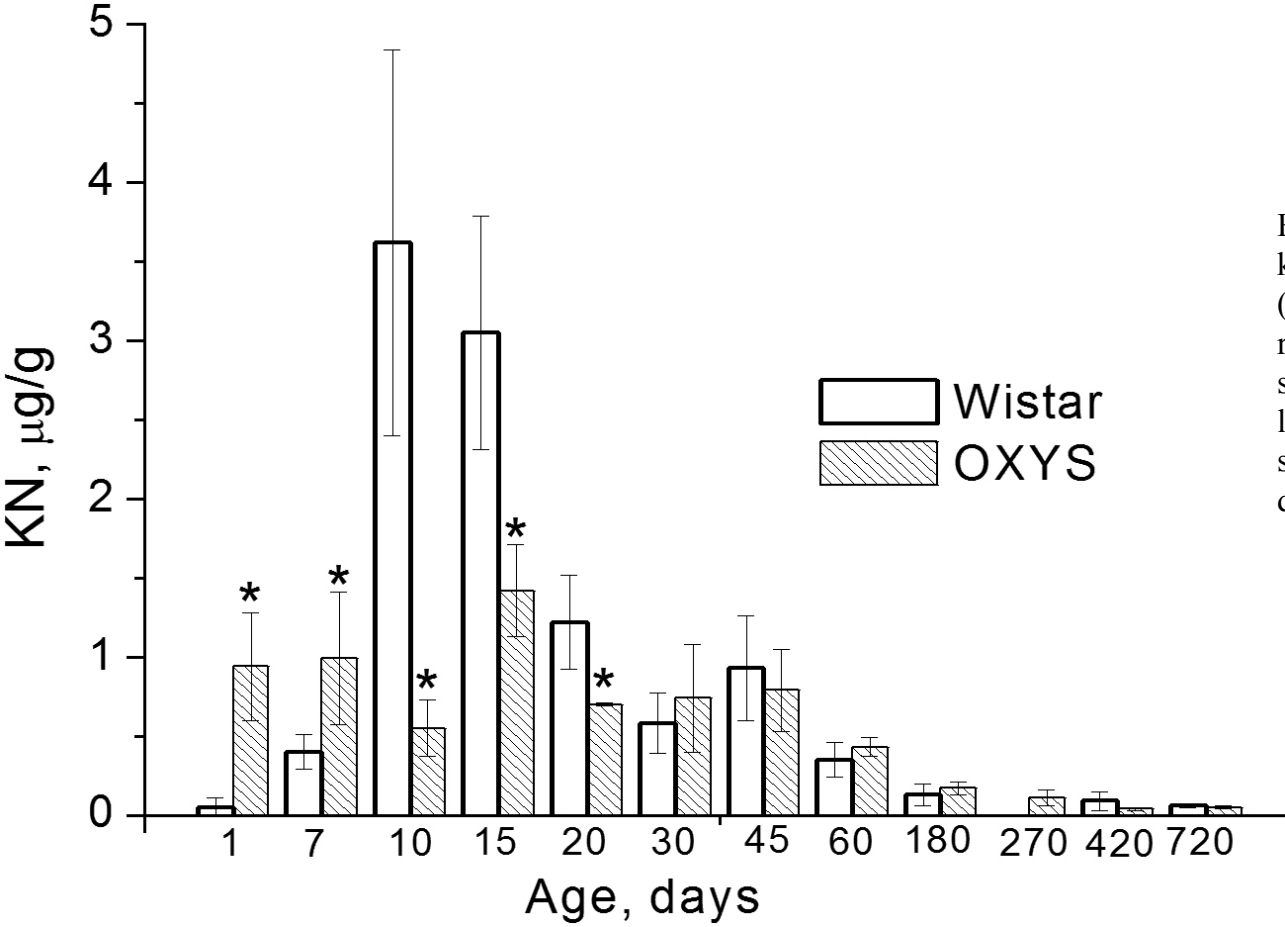
Depicted is the content of kynurenine (KN) in lenses of Wistar (white bars) and OXYS (dashed bars) rats of different age. Error bars show standard deviations of KN level in lenses. The asterisk indicates statistically significant interstrain differences.

The levels of KN and Trp in the lens extracts were quantified using the calibration by solutions of standard KN and Trp. The HPLC measurements were performed for a total of 63 OXYS lenses and 57 Wistar lenses; the age of animals varied from 1 day to 2 years. Typically for every age, the lenses from four to six OXYS and four to six Wistar rats were taken. The concentrations of KN and Trp in each pair of lenses were measured separately, and the data obtained for the same strain and the same age were averaged. The results are presented in Figure 2 and Figure 3, the data obtained for individual rats are given in Table 1.

**Table 1:** Levels of kynurenine and tryptophan in the rat lenses.

			**Level, μg/g**					**Level, μg/g**	
**Strain**	**Age, days**	**Weight of lens pair, mg**	**Tryptophan**	**Kynurenine**	**Strain**	**Age, days**	**Weight of lens pair, mg**	**Tryptophan**	**Kynurenine**
Wistar	1	7.8	9.4	0.08	OXYS	1	6.0	223.4	0.45
		6.4	12.2	0.07			5.0	376.7	0.73
		7.5	10.7	0.13			5.6	289.2	0.78
		7.0	46.7	0			4.3	362.1	0.63
		5.8	39.9	0			6.7	180.3	1.14
							5.6	207.5	1.44
		Average	27.4±18.6	0.05±0.06			Average	283.2±88.5	0.94±0.34
Wistar	7	15.1	66.6	0.26	OXYS	7	11.2	63.1	0.69
		14.1	67.1	0.30			12.6	75.2	0.88
		11.6	87.4	0.45			12.0	173.4	0.94
		14.4	80.3	0.58			17.0	150.4	1.70
		15.5	49.5	0.35			12.1	163.4	1.23
		16.3	63.1	0.36			16.1	167.8	0.50
		15.2	79.6	0.49					
		Average	70.5±12.8	0.40±0.11			Average	132.2±49.6	0.99±0.42
Wistar	10	25.6	42.6	2.56	OXYS	10	20.5	122.1	0.481
		18.8	70.9	3.07			19.6	143.9	0.447
		22.2	64.6	5.40			19.2	180.2	0.297
		26.5	35.3	2.46			17.0	207.1	0.637
		19.8	47.0	3.38			17.8	181.8	0.655
		27.6	55.0	4.84			19.1	196.9	0.804
		Average	52.6±13.5	3.62±1.22			Average	172.0±32.5	0.55±0.18
Wistar	15	14.9	61.6	2.53	OXYS	15	25.0	113.8	1.15
		29.0	45.1	3.04			22.3	111.1	1.18
		18.2	64.0	2.16			21.4	99.2	1.26
		26.3	63.1	3.80			33.2	97.1	1.58
		22.7	40.7	2.68			21.2	125.8	1.91
		19.6	72.8	4.07			38.8	59.3	1.41
		Average	57.9±12.3	3.05±0.74			Average	101.1±23.0	1.42±0.29
Wistar	20	33.6	33.8	1.00	OXYS	20	21.7	108.6	0.71
		31.9	48.2	1.43			25.4	100.4	0.70
		Average	41.0±10.2	1.22±0.30			Average	104.5±5.8	0.70±0.01
Wistar	30	44.9	33.0	0.69	OXYS	30	39.2	43.9	0.41
		40.1	55.3	0.36			43.2	51.1	0.51
		43.3	41.5	0.77			42.6	51.5	1.18
		42.6	49.6	0.78			43.5	59.8	0.53
		45.2	37.0	0.36			41.5	56.2	0.43
		41.1	48.9	0.52			42.0	51.0	1.08
							41.1	44.5	1.03
		Average	44.2±8.5	0.58±0.19			Average	51.1±5.7	0.74±0.34
Wistar	45	51.7	33.6	1.37	OXYS	45	43.0	50.5	0.91
		44.6	33.8	0.91			57.4	44.8	0.70
		53.6	22.9	1.03			56.0	39.7	0.73
		49.6	33.1	1.41			49.2	35.9	1.02
		51.6	29.2	0.87			53.6	37.6	0.95
		50.1	33.6	0.64			50.2	36.8	1.24
		45.5	28.8	0.67			50.0	28.7	0.95
		53.5	35.5	0.52			52.2	45.7	0.43
							49.2	45.5	0.50
							51.7	46.3	0.52
		Average	31.4±4.1	0.93±0.33			Average	41.1±6.5	0.79±0.26
Wistar	60	61.1	33.4	0.57	OXYS	60	65.6	21.3	0.34
		35.8	22.6	0.24			51.7	30.3	0.45
		63.2	20.1	0.40			52.6	30.9	0.43
		49.0	23.4	0.23			58.3	25.0	0.38
		54.8	34.0	0.39			54.8	37.6	0.42
		52.3	24.7	0.30			54.1	40.67	0.53
		57.0	34.8	0.32					
		Average	27.6±6.2	0.35±0.12			Average	31.0±7.3	0.43±0.06
Wistar	180	104.7	26.1	0.15	OXYS	180	106.4	31.3	0.13
		130.0	26.5	0.07			101.3	37.2	0.15
		107.5	27.5	0.09			92.3	29.4	0.16
		108.2	33.4	0.22			93.5	27.5	0.22
		Average	28.3±3.4	0.13±0.07			Average	31.4±4.2	0.17±0.04
					OXYS	270	115.3	24.9	0.06
							118.4	25.2	0.14
							117.2	35.9	0.08
							124.7	27.8	0.17
							Average	28.5±5.1	0.11±0.5
Wistar	420	157.3	13.2	0.18	OXYS	420	150.4	17.4	0.04
		155.5	20.6	0.04			153.7	20.8	0.05
		150.4	22.2	0.07			130.8	21.7	0.05
		138.3	24.1	0.06			138.7	16.2	0.03
		Average	20.0±4.6	0.09±0.06			Average	19.0±2.6	0.04±0.01
Wistar	720	146.6	42.5	0.07	OXYS	720	145.0	40.8	0.07
		155.0	32.3	0.05			154.4	40.4	0.04
							141.4	28.3	0.04
							165.0	38.7	0.05
		Average	37.4±7.2	0.06±0.1			Average	37.1±5.9	0.05±0.1

The concentrations of KN and Trp in each pair of lenses were measured separately, and the data obtained for the same strain and same age were averaged.

Lenses from young and old animals significantly differ in size (Table 1), so it is important to ensure that the employed method of lens treatment provides the extraction of major amounts of KN and Trp independent of the lens size. In a control experiment, we performed three consecutive extractions from several lenses of different sizes (the lens weights varied from 17 to 70 mg); the level of KN and Trp in every extract was measured separately. The typical ratio of the chromophore amounts in extracts was 1:0.2:0.07 and for the most part did not depend on the lens size. Thus, the employed method (two consecutive extractions followed by extract pooling) provides more than 90% extraction of compounds under study from the lens homogenates.

The obtained experimental data were subjected to dispersion analysis. Two-way ANOVA  revealed the effects of both factors, age (F_10,108_=29.8, p<0.0000) and genotype (F_1,108_=100.4, p<0.0000), on the Trp level in the lens of an animal. At the same time, the factors age and strain showed an inter-relationship (F_10,108_=24.9, p<0.0000), which points to a difference in age-related changes in OXYS and Wistar rats. The intra-strain analysis showed that the Trp level in Wistar lenses increased by a factor of 3 (p<0.0001) during the first week of life and reached its maximal value. Afterward, the Trp concentration in Wistar lenses gradually decreased with age, reaching 40% of the maximal value at the age of 2 months (p<0.0001), and then remained at this level up to the age of 2 years.

An extremely high Trp level was revealed in newborn OXYS rat lenses, which had a Trp concentration an order of magnitude higher than in Wistar lenses. During the first 20 days, the Trp level in OXYS lenses remained noticeably higher than in Wistar lenses; however, in rats older than 1 month the difference in Trp concentrations between OXYS and Wistar strains became statistically insignificant. Similar to the Wistar rats, the Trp concentration in OXYS rat lenses decreased during the first 2 months and then remained at a relatively constant level.

The dynamics of KN concentration in Wistar and OXYS lenses are more complex than that of Trp. The KN level in rat lenses depends on both age (F_10,108_=37.5, p<0.0000) and genotype (F_1,108_=18.4, p<0.0000). The interaction of age and genotype (F_10,108_=22.9, р<0.0000) indicates that the age-related changes of the KN level in OXYS and Wistar rats differ. In OXYS lenses, the KN concentration remained almost age independent during the first 1.5 months, at the level of approximately 1 μg/g, and then gradually decreased below 0.1 μg/g. The KN level in Wistar lenses of 7-day-old (0.4 μg/g) and especially in newborn (0.05 μg/g) rats was much lower than in the OXYS lenses of the same age; however, during the first 10 days the KN concentration in Wistar lenses steeply increased and then gradually decreased. At the ages of 10 days, 15 days, and 20 days, the KN level in Wistar lenses was higher than in OXYS lenses by factors of 6.6, 2.1, and 1.7, respectively. In lenses of 1 month and older, the difference between strains is statistically unreliable.

## Discussion

In mammalian cells, the essential amino acid tryptophan degrades primarily by the KN pathway, a cascade of enzymatic steps involving several biologically active compounds. The reaction products, KN and its derivatives, have been shown to be involved not only in a variety of physiological functions but also in many diseases, including cataract [37]. It is well documented that a deficiency of Trp in the rodent diet causes cataract development [36,38,39]. However, there are scarce data on the level of KN in the rodent lens and on the correlation between the KN content and cataractogenesis in experimental animals. Here we provide the evidence that KN is present in the rat lens, although its concentration (especially in adult animals) is low.

The present study demonstrates that the content of Trp and KN undergoes dramatic and rapid changes in the lens during postnatal development of both Wistar and OXYS rats. In the Wistar rat lens, the maximal concentrations of Trp and KN are reached at 7–10 and 10–15 days, respectively, of postnatal development, while in the OXYS lens relatively high concentrations of Trp and KN are found in newborn animals. The difference between the contents of Trp and KN in Wistar and OXYS lenses remains statistically significant during the first 20 days after birth.

It is interesting to compare the dynamics of the evolution of Trp and KN concentration with the progress of eye maturation. The first postnatal 20 days is an important period in lens development. During this period, atrophy of a supporting vascular bed tissue takes place as well as the elimination of potentially light-scattering intracellular organelles in the lens cells, including nuclei and all associated nucleic acids. This process is a key feature of the differentiation of lens epithelial cells into fiber cells and is thought to involve at least some components of the apoptosis signaling pathway [40,41]. The administration of cataractogenic agents is only effective during the first 21 days of rat lens development, a period of the highest sensitivity of the tissue [42].

The transformation of Trp into KN is mediated by indoleamine 2,3-dioxygenase (IDO). The activity of this enzyme may alter the local trp concentration and the build-up of KN pathway metabolites. It has been recently reported that IDO overexpression causes posttranslational modifications of the lens proteins, fiber cell apoptosis, and acceleration of cataract development [35]. We did not study the activity of this enzyme; however, our results show that during the critical period of the lens development (1^st^ to 20^th^ day), the Trp:KN ratio in OXYS lenses is shifted toward Trp. Perhaps this observation is an indirect indication of reduced IDO activity and of the imbalance in the KN pathway in general.

The dynamics of Trp and KN contents in the lens can also be compared with cataract development in the rats under study [33]. The first minor changes in Wistar lenses appear at the age of 6 months; approximately 5% of the rats have weak lens opacity. At the age of 12 and 24 months, 20 and 80% of animals, respectively, have cataract. Changes in eyes of 2-year-old Wistar rats correspond to the initial stage of cataract (very light cortical or nuclear opacity in the lens). Pathological changes in OXYS rat lenses develop much faster: 20% of 45-day-old animals have initial signs of cataract; at the age of 3 months, changes in lenses are found in 90% of OXYS rats; and in 6-month-old animals, morbidity reaches 100%. At the age of 12–14 months, the cataract in OXYS rats reaches stages suggesting significant deterioration in the acuteness of vision. Thus, the manifestation of initial stages of cataract appears in OXYS rats in the period from 1.5 to 3 months when the interstrain difference between the contents of Trp and KN in Wistar and OXYS lenses becomes insignificant.

The amount of KN found in rat lenses is far too low to provide a UV-filtering function. In normal human lenses, the total concentration of UV filters can be estimated [19] as approximately 500 nmol/g; the typical absorption coefficients of these molecules at the absorption maximum (360 nm) are about 5×10^3^ 1/M cm. Such concentrations of UV filters provide an absorption of 90% of UV-A light reaching the lens. In rat lenses, KN was the only UV filter found in the present study and its concentration is two orders of magnitude lower than the total concentration of UV filters in the human lens.

In conclusion, the presented results demonstrate that the KN pathway of Trp catabolism does not play a significant role in cataract development in the rat lens at the stages of cataract manifestation. In the period of 1.5–6 months, when the difference in cataract development in Wistar and OXYS rats is the most pronounced, the interstrain difference in Trp and KN levels is insignificant. However, in the first 3 weeks of postnatal development, this difference is very strong, which demonstrates the correlation between Trp and KN levels at the stage of lens maturation and future cataractogenesis. This suggests that an imbalance in the KN pathway at early stages can create a metabolic background for future cataract development.
